# Phonological Awareness and Rapid Automatized Naming Are Independent Phonological Competencies With Specific Impacts on Word Reading and Spelling: An Intervention Study

**DOI:** 10.3389/fpsyg.2018.00320

**Published:** 2018-03-13

**Authors:** Caroline Vander Stappen, Marie Van Reybroeck

**Affiliations:** Psychological Sciences Research Institute, Université catholique de Louvain, Louvain-la-Neuve, Belgium

**Keywords:** phonological awareness, rapid automatized naming, reading, spelling, interventions

## Abstract

Phonological awareness (PA) and rapid automatized naming (RAN) have been shown to be powerful predictors of reading achievement across many languages. However, literature remains unclear: (a) whether RAN is independent of PA, (b) about the specific influences of PA and RAN on reading and spelling, and (c) about the efficacy of a RAN intervention. This study aims to address these issues by means of an intervention design. Precisely, the objectives are (a) to determine whether training one competence involves or not an effect on the other, (b) to examine whether each intervention based on oral abilities (PA vs. RAN) could improve word reading and word spelling performances, and (c) to assess the efficacy of a RAN-objects’ intervention. Thirty-six French-speaking second graders, from two Belgian elementary schools, were divided into two groups, and received either a PA- or a RAN-objects’ intervention. Twenty-five-minute lessons took place at school twice a week over a period of 2 months. Both groups were compared on multiple experimental measures (PA, RAN, word reading, and word spelling), before and immediately after the intervention, and 6 months later. Results showed specific efficacy of the two interventions, with participants trained in one ability outperforming those from the other group on this specific ability at post-test. Moreover, the PA intervention revealed transfer effects on the sub-lexical processes of spelling, while the RAN intervention enhanced word reading speed. Finally, the results demonstrated the efficacy of a RAN-objects’ intervention for the first time. These findings provide a new piece of evidence showing the independence of PA and RAN, each process influencing the acquisition of literacy skills in a different way. The efficacy and the specific transfer effects of both interventions open up new perspectives for prevention and targeted remediation of reading disabilities.

## Introduction

Mastering literacy skills is a necessary ability for people’s social, educational, and professional development. Research on the precursors of reading is of great importance in grasping the nature of the underlying cognitive processes in reading. Moreover, they constitute malleable fertile ground, on which professionals can work in pre-readers to facilitate literacy development and prevent learning disabilities. As differences between pupils in terms of reading and spelling achievement are partly explained by these precursors, they are already noticeable prior to the onset of formal instruction (Puolakanaho et al., 2007). Among them, phonological awareness (PA) and rapid automatized naming (RAN) are well established as having a major impact on literacy acquisition in a lot of alphabetic writing systems varying in orthographic consistency (e.g., Vaessen and Blomert, 2010; Ziegler et al., 2010; Caravolas et al., 2012; Moll et al., 2014; see also Scarborough, 1998 for a meta-analysis). They are also among the most robust correlates of reading difficulties (e.g., Pennington and Lefly, 2001; Ho et al., 2002; Landerl et al., 2013; Torppa et al., 2013). However, several points remain unclear regarding the relationship between PA, RAN, and literacy skills. A first critical issue concerns how separable RAN is from PA. Some authors argue that they should be considered as a whole, under the phonological umbrella. Others claim that RAN implies cognitive processes that are independent from PA, and should be considered separately from the phonological family. So far, studies have mainly examined this question through correlations, whereas an intervention design would be more powerful in addressing this question. Second, there is no consensus regarding the specific contribution of PA and RAN to literacy development. Results are mixed and no research compared their respective influence on the different subcomponents of literacy. Third, literature typically focused on the impact of PA training, which is widely recognized as being an efficient way to reduce the incidence of or to treat reading and spelling deficits. However, it is still unclear whether it is possible to enhance RAN, and whether it can provide benefits in reading or spelling.

The goal of this study is to investigate these three gaps by means of an intervention design. We want to compare the efficacy of a PA and a RAN intervention, as well as to examine their specific transfer effects on word reading and spelling. This should enable us to add strong empirical arguments for their dependence/independence. Furthermore, highlighting the efficacy of a RAN intervention, and observing the precise causal contribution of each intervention to word reading and spelling could guide the development of new kinds of targeted interventions for disabled readers.

### A Phonological Family or Independent Predictors of Reading?

Phonological awareness is commonly defined as the ability to identify and consciously manipulate the sound units of language. The PA–reading relationship has been extensively studied. For years, research has promoted the idea that an explicit awareness of the phonemes is crucial for reading development (Liberman et al., 1974; Morais et al., 1987). Becoming aware that the flow of speech is actually composed of multiple sounds is a primary foundation, with letter knowledge that makes the child able to discover that each sound is associated with a letter or a letter cluster (i.e., the alphabetic principle; Byrne, 1998). PA, as well as explicit letter-sound instruction, is thought to be the foundation of phonological recoding and of the self-teaching hypothesis (Share, 1995).

RAN refers to the ability to name as quickly as possible an array of highly familiar visual stimuli presented on one page (Kirby et al., 2010). Research on RAN began with the work of Denckla and Rudel (1974, 1976a,b), who developed timed naming tests called *Rapid Automatized Naming* with two types of stimuli: either alphanumeric (digits and letters) or non-alphanumeric (colors and objects). They wanted to test, with young readers, Geschwind and Fusillo’s (1966) hypothesis, which stipulates that poor performances in naming tests could reflect a visual–verbal disconnection explaining the reading difficulties. They found that naming speed was more predictive of reading disabilities than naming accuracy. Since then, many studies have shown that RAN is associated with literacy development, especially with reading speed (for a review, see Georgiou and Parrila, 2013). As an explanation of the RAN–reading relationship, RAN has been initially recognized as a measure of the speed of access to stored phonological information in long-term memory (Torgesen et al., 1997). However, other theoretical explanations currently coexist (i.e., an orthographic interpretation: Bowers and Wolf, 1993; Bowers and Newby-Clark, 2002; a more integrative view: Wolf and Bowers, 1999; Norton and Wolf, 2012) and the reason why RAN is related to reading is still debated.

Phonological awareness, RAN, and also phonological short-term memory have long been considered as part of a whole, *the phonological family*. This traditional view of a triad dates back to the work of Wagner and Torgesen (1987) who observed systematic deficits in these three phonological abilities in children with dyslexia, suggesting a *core phonological deficit* of dyslexia. From that time, evidence from a wide range of studies had supported the idea that poor readers were struggling with PA and with other tasks requiring the processing of phonological information (Wagner and Torgesen, 1987; Rack et al., 1992; Szenkovitz and Ramus, 2005; Melby-Lervåg et al., 2012). In this line of research, PA and RAN are recognized as related subcomponents, contributing together to the prediction of reading skills. In poor readers, RAN deficits are considered as a manifestation of phonological problems. In a review, Vukovic and Siegel (2006) cited three arguments in favor of PA-RAN dependence: PA and RAN are highly correlated; it is difficult to find children with a RAN deficit without a concurrent PA deficit; some PA interventions have been found to reduce the occurrence of RAN deficits. Some studies using mediation analyses also subsume RAN under phonological processing. Bowey et al. (2005) observed that the impact of RAN on reading was partially mediated by PA in normal Grade 4 readers. In the same vein, Poulsen et al. (2015) conducted a large-scale longitudinal study assessing PA and RAN in kindergarten and reading outcomes in Grade 1. They found that PA and letter knowledge explained from 18 to 56% of the RAN–reading relationship.

Beside the phonological family hypothesis, some researchers suggested that RAN could be another cause of developmental dyslexia, separate from the phonological family. This hypothesis takes inspiration from the observation that some children experience reading difficulties despite good performance in PA and that phonologically based interventions are not always sufficient to improve reading skills (Bus and van Ijzendoorn, 1999; Torgesen, 2000). Wolf and Bowers (1999) suggested that naming speed should be considered as a second core deficit among children with dyslexia. They proposed *the double-deficit hypothesis* (DDH), where PA and RAN were depicted as two separable sources of reading dysfunction. According to this hypothesis, four groups can be predicted among young readers: children with no-deficit (double-asset group), children with a single deficit in PA, children with a single deficit in RAN, or children with a double-deficit (in both PA and RAN). The latter would experience more widespread reading difficulties compared to children with a single deficit. This hypothesis has been studied extensively over the last years. Many studies have found such profiles of readers in different languages (e.g., Portuguese: Araújo et al., 2010; Spanish: López-Escribano, 2007; Greek: Papadopoulos et al., 2009; Finnish: Torppa et al., 2013; English: Wolf et al., 2002). Even a recent neuroimaging study supported this view (Norton et al., 2014). PA training studies in children with dyslexia also suggest that RAN is separated from PA. First, Lovett et al. (2000) observed that all reading-disabled children benefited from their phonological intervention but that children with a single PA deficit exhibited significantly greater gains in reading than children with a single RAN deficit or with the double deficit. Second, PA training studies in which RAN was measured did not find any improvement of the RAN times at post-test (Lovett et al., 2000; Regtvoort and van der Leij, 2007). In typically developing children, several studies showed that PA and RAN were poorly related (see the meta-analysis of Swanson et al., 2003) and that they loaded on different factors (Swanson et al., 2003; Lonigan et al., 2009). Similarly, Powell et al. (2007) demonstrated that a SEM model in which PA and RAN were separated predictors of reading provided a more convincing fit to the data than two other models where PA was subsumed within a phonological processing factor. By performing hierarchical regression analyses, other researchers found that RAN contributed to reading skills beyond the contribution of PA (Manis et al., 1999, 2000; Clarke et al., 2005).

To summarize, there are still two opposing conceptualizations of the PA–RAN relationship. Studies in pathology and in normal readers led to opposite conclusions. They did not establish whether PA and RAN are independent skills or not. Besides, it is noteworthy that most existing research is correlational in nature. To date, directly manipulating both variables through interventions to study their interactions has not been done. The current study proposes to compare the direct effects of a PA intervention and a RAN intervention on PA and RAN in order to provide causal rather than merely correlational evidence of the independence of these abilities (objective 1).

### Specific Contributions of PA and RAN to Literacy Skills

The existing literature provides evidence of some specific contributions of PA and RAN to literacy development. On the one hand, PA is thought to generally impact accuracy of word identification, and more specifically non-word reading accuracy in typically developing children (Badian, 1993; Manis et al., 1999, 2000; Swanson et al., 2003) and in dyslexic readers (Griffiths and Snowling, 2002; Sunseth and Greig Bowers, 2002). Indeed, the ability to read unfamiliar words critically depends on segmental abilities (Morais et al., 1987). Their decoding requires individual manipulations of the phonemes. However, PA does not seem to contribute to the prediction of reading speed (Landerl and Wimmer, 2008; Fricke et al., 2016). Moreover, across different orthographies (English, Norwegian, Swedish, and Chinese), PA seems to be more related to spelling than reading (Landerl and Wimmer, 2008; Furnes and Samuelsson, 2011; Pan et al., 2011; Wolff, 2014). Two studies respectively conducted in German (Wimmer and Mayringer, 2002) and in Finnish (Torppa et al., 2016) found that a single reading fluency deficit was not associated with phonological deficits, whereas a single spelling deficit was. Schneider et al. (2000) found more pronounced transfer effect from the PA training for spelling than for reading. Furthermore, it has been demonstrated that some specific spelling errors (i.e., omissions in consonant clusters) committed by children with dyslexia and by children who are learning to spell directly reflect a poor ability to identify the phonemes in spoken words (i.e., spoken words including a consonant cluster; Bruck and Treiman, 1990). Phoneme awareness seems to be particularly crucial for spelling development. On the other hand, the majority of evidence has revealed that RAN did not exert any significant direct effect on spelling (Wolff, 2014; Georgiou et al., 2016). Longitudinal studies rather found RAN as the most important predictor of reading measures (Landerl and Wimmer, 2008; Furnes and Samuelsson, 2011). In poor readers, some authors demonstrated that poor performance in RAN predicted dysfluent reading, but not spelling deficits (Wimmer and Mayringer, 2002; Torppa et al., 2016). Furthermore, RAN was found to contribute even more to reading speed than to reading accuracy across numerous languages (van den Bos et al., 2002; Georgiou et al., 2016; see also Araújo et al., 2015 for a recent meta-analysis). Therefore, RAN has been identified as a universal marker of reading speed through automaticity.

Taken together, these observations suggest that PA and RAN differ in their respective predictive weight of the literacy subcomponents, but there is a critical methodological limitation in the pre-cited studies. The majority of the authors studying the RAN–reading relationship mainly used a single reading fluency measure without any measure of reading accuracy. However, a longitudinal study conducted among Grade 2 German children showed that preschool RAN significantly contributed to both reading speed and accuracy (Fricke et al., 2016). Moreover, some findings are inconsistent. Indeed, RAN has sometimes been found to substantially predict spelling (Savage et al., 2008). Finally, no study has directly examined the specific influences of PA and RAN on word reading (accuracy vs. speed) and spelling concomitantly within an intervention design (objective 2).

### RAN Intervention Studies

An abundance of studies highlighted the causal impact of PA training on reading performance (for meta-analyses, see Bus and van Ijzendoorn, 1999; Ehri et al., 2001). More recently, Hulme et al. (2012) provided strong support to PA instruction and its benefit in the long run. They causally demonstrated the efficacy of a phoneme awareness and letter-sound knowledge intervention with mediation analyses and path modeling, which captured the dynamics between all variables. Letter-sound knowledge and phoneme awareness outcomes measured at the end of the intervention fully mediated the literacy improvements noticeable 5 months later.

While it is now well established that training PA remarkably improves children’s ability to read and to spell, RAN is often thought to be impossible to train. Indeed, authors have failed to give evidence in favor of the efficacy of a RAN training until now. To the best of our knowledge, only four studies have examined how RAN performance can be improved, and they all involve some limitations. First of all, only one of them concerned training the RAN objects (Eleveld, 2005, Unpublished), with the three others only focusing on letter-naming speed (Fugate, 1997; de Jong and Vrielink, 2004; Conrad and Levy, 2011). This led to confusing interpretations, presumably because they did not target exactly the same processes. Naming letters is quite similar to word decoding, causing a certain circularity in the interpretation. Besides, RAN letters could reflect the access to a code, while RAN objects require the retrieval of a lexical unit. Second, the four studies found mixed and unconvincing results. Fugate (1997) found a significant effect of letter-naming speed training in Grade 1 children at post-test, with improvement of the letter-naming speed and a small subsequent increase in reading fluency. However, these effects were no longer significant at follow-up, and intervention transfer to reading was only shown for one of the four reading measures. In de Jong and Vrielink (2004), the children trained in letter-sound naming speed did not outperform the non-trained group on this measure following the intervention. Conrad and Levy (2011) conducted crossover interventions in orthographic pattern recognition and fast letter recognition with Grade 1 and Grade 2 children who had poor reading scores and slow naming speeds. Results showed that training in speeded letter recognition provided benefits in speeded letter recognition but only when preceded by the training in orthographic pattern recognition. However, the combination of the two consecutive interventions could have led to confounding training effects. Third, it should be noticed that the training did not always include matrices (serial RAN) but sometimes corresponded to a flashcard drill of individual letters (Fugate, 1997; de Jong and Vrielink, 2004). Yet, the literature shows that serial RAN is more strongly associated with reading than discrete RAN (Wolf and Bowers, 1999; Logan et al., 2011). Finally, some authors combined their intervention with a drill of other abilities as letter-to-sound rules (de Jong and Vrielink, 2004) or PA skills (Eleveld, 2005, Unpublished). As a consequence, RAN training effects were confounded with other effects.

To conclude, PA has long been considered a primary starting point for remediation as well as a means of preventing reading difficulties from occurring, while the clinical implications of RAN have not received the same acknowledgment. There is currently no strong evidence for the efficacy of RAN training and its potential benefits for literacy skills. Furthermore, no research has tested this hypothesis with “pure” RAN-objects’ training. Consequently, an important aim of this study is to evaluate the efficacy of a RAN-objects’ intervention (objective 3).

### The Present Study

Reviewing the literature related to PA, RAN, and literacy led us to identify several gaps that require further investigation. The main aim of the current study is to determine for the first time whether RAN is independent of PA through an intervention study (objective 1). Examining the specific efficacy of a PA intervention and a RAN intervention will enable us to add strong experimental arguments of their dependence/independence beyond the correlation evidence provided by earlier studies. According to the independence hypothesis, we would expect that children trained in PA will improve in PA and not in RAN, and that children trained in RAN will be better in RAN but not in PA after the intervention. According to the dependence hypothesis, both interventions would induce effects on both PA and RAN outcomes.

Then, discrepancies exist and there is no consensus about the specific contribution of PA and RAN to the subcomponents of literacy. A second purpose of this study is to find out the specific causal influence of RAN and PA on word reading and spelling by examining the transfer effects of the interventions (objective 2). We expect that the PA intervention will give an advantage to reading and spelling accuracy, with a greater impact on spelling. Conversely, we anticipate the RAN intervention to be beneficial in terms of reading speed.

Finally, there has been no strong evidence of the efficacy of RAN training until now. Therefore, we wanted to examine if it is possible to train the RAN procedure (objective 3). This has never been demonstrated with serial RAN objects alone. We chose to target RAN objects because we wanted to measure the implication of direct access to the phonological representations of real lexical units (i.e., entire words), rather than access to a code such as in RAN digits and in RAN letters.

## Materials and Methods

### Participants

Four Grade 2 classes from two Belgian elementary schools took part in the experiment. One school (school 1) comes from a middle socio-economic area whereas the other school (school 2) is from a high socio-economic area. Both are located in the French-speaking part of Belgium and advocate a phonics-based method of learning to read.

All the children from the selected classes (*N* = 56) were invited to participate in the experiment. Five children, whose parents did not give their consent, received the intervention without being administrated the pre- and post-tests’ measures. Therefore, they were not included in our sample. Moreover, 15 children who also took part in the intervention were removed from the analyses because they met one of the exclusion criteria: (a) three children scored below two standard deviations for their age in non-verbal IQ [as measured by the matrices’ subtest of the WISC-IV (fourth edition of the Wechsler Intelligence Scale for Children); Wechsler, 2005]; (b) four children scored below two standard deviations for their age in productive vocabulary (pictures’ naming of the ELO; Khomsi, 2001); (c) five children had been receiving speech therapy for language impairment or difficulties with written language; and (d) three children had dropped out at follow-up because they moved to another school. The final studied sample was made up of 36 participants who were randomly assigned to one of the intervention groups. In each class, a RAN subgroup (receiving a RAN intervention) and a PA subgroup (receiving a PA intervention) were formed, resulting in eight intervention subgroups. This was done to control for possible teacher effect. The proportion of participants from the two schools was equivalent in both intervention groups. Therefore, the groups are matched on socio-economic status. **Table 1** shows the distribution of participants from the two schools by intervention group.

**Table 1:** Number of participants assigned to each intervention.

		School 1			School 2
	*N*	Class 1	Class 2	Class 3	Class 4
PA intervention	18	3	5	4	6
RAN intervention	18	4	5	3	6

*PA, phonological awareness; RAN, rapid automatized naming.*

Eighteen participants received the PA intervention (11 males; *M*_age_ ±*SD*: 7.53 ± 0.30 years), and 18 others received the RAN intervention (six males; *M*_age_ ±*SD*: 7.48 ± 0.33 years). The mean age of the children was 7.50 years (*SD* = 0.31). The characteristics of the children on the control measures (non-verbal IQ, vocabulary), their PA and RAN outcomes at pre-test, as well as their chronological age are reported in **Table 2**. Independent samples’ *t*-tests were conducted on theses variables, with group as a between-participant factor to ensure that both experimental groups were equivalent prior to the interventions. Although no significant differences were found across groups (*p*s > 0.07, **Table 2**), the group effect on RAN showed a *p*-value reaching almost the significant threshold, and a Cohen’s *d* indicating a moderate effect, *t*(34) = -1.89, *p* = 0.07, *d* = -0.65 (Cohen, 1988). This has been taken into account in the analyses.

**Table 2:** Participants’ characteristics and equivalence between intervention group at pre-test using *t*-test analyses.

Measures	PA intervention (*N* = 18)		RAN intervention (*N* = 18)		Group effects		
	*M*	*SD*	*M*	*SD*	*t*(34)	*p*	Cohen’s *d*
Age (years)	7.53	0.30	7.48	0.33	-0.47	0.64	-0.16
Non-verbal IQ (scaled score)^a^	9.67	2.83	10.28	2.56	0.68	0.50	0.23
Vocabulary (raw score)^b^	33.11	4.97	33.61	3.52	0.50	0.62	0.17
Phonological awareness (raw score)^c^	25.39	7.23	25.39	6.16	0.00	1	0.00
RAN (composite score)^d^	1.15	0.27	0.99	0.24	-1.89	0.07	-0.65

*^a^Matrices’ subtest of the WISC-IV, M = 10, SD = 3. ^b^Pictures’ naming of the ELO, total correct responses out of 42. ^c^Syllable and phoneme deletion task from the battery for the assessment of phonological skills, total correct responses out of 35. ^d^RAN composite score, total correct responses divided by total naming time.*

The study was approved by the Ethics Committee of the Psychological Science Research Institute (approval number 2014-37), and was carried out with written informed consent from all participants’ parents. The authors declare that they complied with ethical standards in accordance with the Declaration of Helsinki.

### Procedure

All testing and interventions took place at school during school hours. In Belgium, the school year runs from September to June. Participants of both intervention groups were assessed and trained by two experimenters (the first author and another researcher). Each child received a battery of tests before (January, Grade 2), immediately after the intervention (April to May, Grade 2), and at follow-up, 6 months later (October, Grade 3). The testing began with a collective administration of the spelling under dictation task. This was given within each classroom over a session lasting around 30 min. Then, the other tasks were conducted individually in a quiet room near the children’s classrooms in a 40-min session. As fatigue was more of a concern than the order effects, the order of the tasks was fixed across participants; the level of difficulty was gradually increasing then decreasing within each session. The standardized tests of non-verbal IQ and vocabulary were administered at the pre-test only. Score sheets were anonymized prior to scoring in order to follow a blind process. The children received either a group-based RAN or PA intervention over a period of 2 months (February to March). There was a 1-week interruption between week 2 and week 3 due to the winter break and a 2-week interruption between weeks 8 and 9 due to the spring break. A total of sixteen 25-min lessons took place twice a week. Each RAN and PA subgroup was made up of seven children (including the participants and the children excluded from the analyses). The training of both subgroups from the same class occurred simultaneously. In order to control for possible experimenter effects, each experimenter took charge of an equivalent number of both intervention subgroups (i.e., two RAN subgroups and two PA subgroups each).

### Intervention Program

A highly structured program was planned carefully with general guidelines: (a) the sequences of exercises gradually increased in level of difficulty; (b) all the children were actively solicited during the lesson; (c) the children were always given several examples before starting an exercise; (d) immediate feedback was systematically given to an answer. If the answer was wrong, the child was first encouraged to self-correct. Then, the other children were invited to help him/her in finding the correct response; (e) all the activities were as fun as possible to maintain the children’s interest. We ensured to control for Hawthorne effects by making both interventions the same structure and length with the same frequency and duration of each lesson.

#### Phonological Awareness Intervention

This intervention was based on the work of Adams et al. (2000) and on the training program of Van Reybroeck et al. (2006a). Those training programs were designed for young children from kindergarten or first grade. Therefore, adaptations were made for Grade 2 children on the basis of the pre-test observations. These revealed that children still experienced particular difficulties in discriminating voiced and voiceless consonants and in identifying and manipulating a phoneme, and particularly when this phoneme was inside a consonant cluster (two or more consecutive consonants). The linguistic material used in the lessons was chosen in order to gradually increase the level of difficulty of the task: (a) the type of items was first familiar words, and then pseudowords; the nature of the target units to be manipulated changed from fricative consonants (f, s, ∫, v, z, 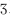) to occlusive consonants (p, t, k, b, d, g); (b) its position varied from the initial position to the final and medial position in the word; and (c) their syllabic structure corresponded first to a simple consonant and then to a consonant which was part of a cluster (CC or CCC). The tasks were also progressively more complex, varying from easy phoneme identification to the most advanced levels of phoneme manipulation. First, the children began with an introductory lesson in which the concept of syllable was revised. They practiced segmenting words into syllables (e.g., /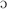
R-di-na-tœR/ for *ordinateur* [computer]), counting syllables (e.g., there are four syllables in *ordinateur*), and identifying syllables (e.g., *ordinateur* includes the syllable /di/). Second, the experimenter told the story of *La Planète des Alphas* ([The Planet of the Alphas]; Huguenin, 1999) to the children and taught them to recognize the different characters (the “Alphas,” corresponding to the letters) and their song (the phonemes). This pedagogical material offers a visual and meaningful support to phoneme representations. Greater efficacy of PA training has been found when using this material rather than simple letters (Van Reybroeck, unpublished). Third, the children learnt to sort words following a specific phoneme (initial, medial, and final) into the right category (voiced or voiceless phoneme). For example, we asked them to determine if the word *table* /tabl/ [table] went rather in the house of the *toupie* ([top], the Alpha character representing the phoneme /t/), or in the house of the *dame* ([lady], the Alpha character representing the phoneme /d/). They also learnt to identify the position of specific phonemes in words (e.g., /d/ is the third phoneme in *ordinateur*). Fourth, the children were taught to segment words/pseudowords into phonemes (e.g., /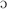-R-d-i-n-a-t-œ-R/), to remove a specific phoneme from a word/pseudoword (e.g., *ordinateur* without the /d/ is *orinateur*), and to remove and blend the first phonemes of two words (e.g., *table* and *ordinateur* → /t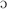/). Finally, they learnt to invert (e.g., arc /aRk/ [bow] → /kRa/) or substitute phonemes in a word (e.g., *ordinateur* →*orbinateur*) and to add phonemes to a word (e.g., /p/ + *ordinateur* →*pordinateur*). None of the activities included any written material. All exercises were done orally. **Table 3** provides an overview of the intervention program.

**Table 3:** Overview of the PA intervention program.

Week	Lesson	PA intervention
1	1	Segmentation of words into syllables and identification of syllables
1–2	2–3	Presentation of the characters of *La planète des Alphas* and practice on recognizing them and their song
2–4	4–7	Phoneme identification in words
4–5	8–9	Segmentation of words into phonemes and identification of phonemes in pseudowords
5–6	10–12	Phoneme deletion and acronyms
7	13–14	Phoneme deletion, acronyms, and phoneme inversion
8	15–16	Mixed activities based on phonemes: deletion, acronyms, inversion, adding, and substitution

#### RAN Intervention

The RAN intervention aimed at increasing the naming speed of objects. Regarding the material, two types of RAN-objects’ matrices were used: RAN-R matrices (RAN repeated) were composed of few items that appeared several times (three items repeated eight times or six items repeated four times) while RAN-NR (RAN non-repeated) included 24 different items. The first eight lessons used RAN-R matrices. The last eight used RAN-NR ones. Two matrices were trained per lesson, resulting in a total of 16 RAN-R matrices and 16 RAN-NR matrices and a total of 192 items trained through the 16 lessons. All items were color pictures of highly familiar objects arranged in a matrix of four lines of six items. Several psycholinguistic variables were controlled. All words were high-frequency nouns, corresponding to the 75th percentile of the word-frequency distribution for Grade 1 children (SFI > 56 in MANULEX; Lété et al., 2004). In addition to the type of RAN matrix, the level of difficulty gradually varied with respect to the length and the complexity of syllabic structure of the words. There were an equivalent number of short (1 syllable, <6 letters) and long (1–4 syllables, 6–12 letters) items, and simple (CV syllable) or complex (CCV syllable) items. **Table 4** provides an overview of the intervention program. Each lesson was constructed in the same way: two RAN matrices were practiced with varied forms of activities. First, every child, one after another, was asked to name as quickly and accurately as possible the objects presented on a RAN matrix. The experimenter timed them. A mechanical ladybird toy, placed on a sloping board, was used to encourage the children to go fast (material from *Lexidéfi*; Bouy, 2003). They had to finish naming the matrix before the ladybird had gone down the board. Second, they were encouraged to do it again more quickly with another RAN matrix to break their own speed record. Third, the same items were practiced in the form of a relay race, in order to make it more active and fun. The objects of both matrices were enlarged and printed in A4 size. They were placed on the ground with a wide space between them. They were arranged either in lines or in a circle. One after another, the children jumped from one item to another, naming them at the same time. They were encouraged to break the previous week’s collective speed record. Finally, the children were asked to draw a matrix from a set including all the matrices already practiced since the beginning of the intervention and to name it quickly. A story with a puzzle to solve was told throughout the 8 weeks to keep the children motivated. They received a piece of the puzzle at the end of each lesson.

**Table 4:** Overview of the RAN intervention program.

Week	Lesson	RAN intervention			
		Type of matrix	Length of words	Syllabic structure	Items’ arrangement
1	1	RAN-R	Short	Simple	3 × 8
	2				6 × 4
2	3			Complex	3 × 8
	4				6 × 4
3	5		Long	Simple	3 × 8
	6				6 × 4
4	7			Complex	3 × 8
	8				6 × 4
5	9	RAN-NR	Short	Simple	
	10				
6	11			Complex	
	12				
7	13		Long	Simple	
	14				
8	15			Complex	
	16				

*RAN-R, rapid automatized naming with repeated items; RAN-NR, rapid automatized naming with non-repeated items; 3 × 8, three items repeated eight times; 6 × 4, six items repeated four times.*

### Treatment Fidelity

To ensure that the interventions were delivered as planned, the following precautions were taken. First, the first author created a manual containing the intervention program with the above-mentioned specific materials and exercises. Both experimenters received practice in applying the training program. Second, a checklist that contained step-by-step directions was provided at each lesson. As they completed a step, the experimenters had to check it off. Examination of the checklists showed that experimenters completed 94.5% of the steps in the PA intervention and 95.14% of the steps in the RAN intervention. Third, the experimenters were asked to take note of the presence of each participant at the 16 lessons. The children took part in 95.14% and 94.74% of the activities, in the PA and RAN groups, respectively. Finally, they met weekly to discuss how the experiment was proceeding in general and any glitches that occurred, to check that the interventions were conducted as similarly as possible within the four subgroups of the same intervention. They also revised the training program according to the children’s improvements to make sure that the activities would neither be too easy nor too difficult to complete. These adjustments were applied at the group level (the same for all children of the intervention group). The children moved onto the next step once the majority of their intervention group had acquired the preceding one. Therefore, the manual and the checklists were updated every week.

### Measures

#### Estimated Non-verbal IQ

The French version of the matrix reasoning subtest from the WISC-IV (Wechsler, 2005) was used to measure non-verbal reasoning. This subtest was identified as a reliable measure of fluid reasoning (Kaufman et al., 2006). It is composed of a series of 35 incomplete matrices containing abstract patterns and designs. The children were required to select the best from one of the five response options in order to complete the matrix. The total number of correct responses (maximum 35) was converted to a subtest scaled score. The reported internal consistency coefficient for this test is 0.86.

#### Vocabulary

The level of productive vocabulary was measured by the pictures naming test of the ELO/Evaluation du Langage Oral ([Oral Language Assessment]; Khomsi, 2001). The children were asked to name a series of 42 pictures, including 32 nouns and 10 action verbs. The maximum raw score is 42. For the current sample, the ELO was found to have an internal consistency coefficient (Cronbach’s α) of 0.78.

#### Phonological Awareness

The syllable and phoneme deletion task from the BEPHO/Batterie d’Evaluation des competences Phonologiques ([Battery for the Assessment of Phonological Skills]; Van Reybroeck, 2003) was adapted by removing 25 items to make a total of 35 items. An equivalent number of items were removed in each category of items from the initial version of 60 items. This was done to prevent fatigue effects during the testing session. First, the task consisted of repeating a non-word pronounced by the experimenter. Second, the children needed to say what would be left after taking away a designated phonological unit, either a syllable or a phoneme. Ten items required the children to remove the initial syllable of a bisyllabic non-word with a CVCV structure (e.g., /m
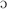
ti/ without the /m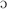/ is /ti/). Twenty-five items required the children to take away a designated phoneme of a unisyllabic non-word. The syllabic structure of the non-word and the position of the phoneme to be removed varied across the items: nine items targeted the initial phoneme of a CVC non-word (e.g., /ky∫/ without the /k/ is /y∫/), six items targeted the initial phoneme of a CCVC non-word (e.g., /pløs/ without the /p/ is /løs/), and 10 items targeted the second phoneme of a CCVC non-word (e.g., /stim/ without the /t/ is /sim/). The different types of items were pseudorandomized in order to prevent guessing. The test was preceded by six practice items to ensure that the children had understood the instructions. The children were given one point for each correct or self-corrected response. The maximum score on the test is 35. The internal reliability (Cronbach’s α) in the current sample is 0.89.

#### Rapid Automatized Naming

Two matrices of objects from the battery for the assessment of phonological skills (Van Reybroeck, 2003) were presented to the participants. All the items were highly familiar French words with an age of acquisition lower than 60 months (Chalard et al., 2003). They were illustrated by color photographs arranged semi-randomly in four rows of six. The matrices were composed of three items repeated eight times (RAN-R matrices). One matrix was made up of short items (one-syllable words) such as *pomme* /p
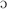
m/ [apple], *clé* /kle/ [key], and *chaise* /∫εz/ [chair]. The other was composed of long items (two- and three-syllables words) such as *guitare* /gitaR/ [guitar], *champignon* /∫ãpi
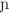

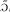
/ [mushroom], and *téléphone* /téléf
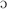
n/ [telephone]. The test matrices were preceded by a training matrix, which included a set of other items to avoid pre-activation of the test items. It should be noted that none of the test items were trained during the intervention. The children had to name as quickly and accurately as possible the objects on the matrix from left to right and from top to bottom. For each matrix, the number of errors and the time to name all of the objects were recorded. A composite score was computed by dividing the number of objects correctly named from the two matrices by the total naming time for both matrices. Thus, a high RAN score indicated a high performance. Reliability analyses returned an alpha coefficient of 0.83 in the current sample.

#### Word Reading

The single word reading task is a standardized test from the BALE/Batterie Analytique du Langage Ecrit ([Analytic Battery of Written Language]; Jacquier-Roux et al., 2010). It consists of two sets of items: high vs. low-frequency words. Each set included three lists of printed words arranged in columns: 20 regular words, 20 irregular words matched on length, and 20 pseudowords, matched on length, phonemic, and orthographic structure with the regular words (Bosse and Valdois, 2009). The high-frequency items were not used in order to avoid a ceiling effect (as expected by the authors of the test as soon as children are in Grade 3). The children were asked to read the items aloud as quickly and accurately as possible. Regarding pseudowords, they were informed that they were not real words and that the purpose was to read them without trying to understand them. Speed (time in seconds) and accuracy (number of items correctly read) were recorded for each list. Reliability analyses returned an alpha coefficient of 0.90 in the current sample.

#### Word Spelling

The spelling under dictation task was composed of three standardized lists of words from the BALE (Jacquier-Roux et al., 2010): a list of 10 complex bisyllabic regular words, another of 10 bisyllabic irregular words, and a last one of 10 trisyllabic pseudowords. The children were asked to spell the single words presented in isolation by the experimenter. The warning in the instructions for pseudowords was similar to that in the reading task (adapted to spelling). The children were given one point for each correctly spelled word, with a maximal score of 10 for each list of words. The internal reliability (Cronbach’s α) in the current sample is 0.72.

### Spelling Errors

Spelling errors were categorized and percentages of the types of errors were computed to allow further investigation of the transfer effect of the interventions on spelling performance. This provided more information about the spelling strategies (i.e., the sub-lexical vs. lexical strategies) the children preferentially used, and the potential changes that could have occurred between the pre-test and either or both of the post-tests. Errors were observed throughout the different spelling lists. Both researchers received the coding of the responses into different categories and were trained with prototypes to analyze them. Spelling errors were categorized into three types.

#### Phonological Errors

These errors were phonologically inappropriate to the target word. They were: (a) omissions such as *vigne* [vine] → vige or *scropal* (pseudoword) → scopal, (b) substitutions such as *poisson* [fish] → pansson or *bracho* (pseudoword) → pracho, (c) additions of a letter or of a letter cluster such as *million* [million] → milieuon or *flocachin* (pseudoword) → flocachien, and (d) serial order errors such as *bleu* [blue] → belu or *abritel* (pseudoword) → arbitel. These errors can be interpreted as a difficulty within the sub-lexical processes.

#### Orthographic Errors

Errors in this category were due to inconsistencies in French orthography. They were all phonologically plausible errors due to: (a) omission of a doubled letter such as *million* [million] → milion, (b) omission of a silent letter such as *serpent* [snake] → serpen, (c) choice of an alternative consonant grapheme for a phoneme such as *peinture* [paint] → pinture, and (d) a lack of knowledge of particular grapheme–phoneme correspondences that are context sensitive, such as *poisson* [fish] → poison (*s* in between two vowels sounds like /z/ instead of /s/) or *siropage* (pseudoword) → siropag (*g* without any vowel following sounds like /g/ instead of /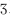/). These errors can be interpreted as indicating a reliance on the sub-lexical procedure because of a lack of lexical knowledge.

#### Non-responses

Errors classified in this category corresponded to an absence of response.

### Statistical Analyses

Statistical analyses were performed using SPSS 22. In a set of preliminary analyses, we first observed the degree of skewness and kurtosis to ensure that the data met the normality assumption of parametric procedures. Their values revealed no distributional problems (skewness < |2| and kurtosis < |7|; Kim, 2013) except for response time (RT) in pseudoword reading. Inspection of the shape of the distributions revealed that RT for other reading variables tended to be asymmetric. For these reasons, and because it is recommended with speed measures, reading RTs were 1/RT transformed in order to reach distributions within acceptable levels of symmetry and peakedness. Second, the group effect on RAN at pre-test being almost significant (**Table 2**), we entered two testing times in the analyses (repeated measures analyses) rather than analyzing the post-tests performances without considering performances at the previous testing time. Third, as participants were nested within intervention subgroups, we tested if there were differences between subgroups for all dependent measures at pre-test. One-way analyses of variance (ANOVAs) using subgroup as a between-participant factor showed no differences across subgroups (*p*s > 0.07), except for pseudoword reading accuracy and regular word reading accuracy. Thus, for these two variables, two-way nested analyses of covariance (ANCOVAs) were conducted on measures at post-test and at follow-up with group as a fixed factor, subgroup as a random factor, and the respective pre-test scores as a covariate. These analyses revealed no effects of subgroup nested within group (*F*s < 1.49, *p*s > 0.22). Therefore, for all the variables, we examined the intervention and transfer effects using repeated measures ANOVAs. Finally, we ensured that our results could not been explained by gender. When repeating our ANOVAs with gender in covariate, we found no significant effect of gender and no significant interactions (Time × Gender, Group × Gender, or Time × Group × Gender).

The intervention effect was analyzed using two-way repeated measures ANOVAs with group as a between-participant factor and testing time as a within-participant factor. Regarding the analyses of the transfer effects, we added a third within-participant factor (three-way repeated measures ANOVAs): word type for reading accuracy and reading speed (regular words, irregular words, and pseudowords) and error type for spelling errors (phonological errors and orthographic errors). These analyses primarily focused on the pre- to post-test results (T1–T2). Then, in order to examine the intervention effect in the long run, additional repeated measures ANOVAs were carried out, including the follow-up in the testing time factor (T1–T2–T3). The assumption of sphericity was checked with Mauchly’s test. We applied Greenhouse–Geisser corrections for data violating the sphericity assumption. The alpha level was set at 0.05 for all the analyses.

## Results

### Efficacy of the Interventions

The efficacy of the interventions was analyzed with two-way repeated measures ANOVAs conducted on PA and RAN. Supplementary Figure 1 reports PA and RAN performances by testing time and group. **Table 5** details mean performances and standard deviations.

**Table 5:** Descriptive statistics on PA and RAN measures by testing time and group.

Measures	Testing time	PA intervention (*N* = 18)		RAN intervention (*N* = 18)	
		*M*	*SD*	*M*	*SD*
Phonological	T1	25.39	7.23	25.39	6.16
awareness	T2	29.39	6.37	25.78	5.26
(raw score)^a^	T3	30.00	4.63	27.89	5.80
Rapid automatized	T1	1.15	0.27	0.99	0.24
naming	T2	1.15	0.20	1.15	0.33
(composite score)^b^	T3	1.28	0.29	1.18	0.27

*T (1–3), testing time. ^a^Syllable and phoneme deletion task from the battery for the assessment of phonological skills, total correct responses out of 35.*

*^b^RAN composite score, total correct responses divided by total naming time.*

#### Phonological Awareness

The 2 × 2 ANOVA showed a significant effect of testing time, indicating that the children from both groups improved in PA from T1 to T2, *F*(1,34) = 8.51, *p* = 0.01, $\eta_{p}^{2}$ = 0.20. The analysis also showed a significant Group × Testing Time interaction, suggesting a better improvement of the children from the PA group, in comparison to the children from the RAN group, from pre-test to post-test, *F*(1,34) = 5.76, *p* = 0.02, $\eta_{p}^{2}$ = 0.15. In addition, including the three testing times (2 × 3 ANOVA), the Group × Testing Time interaction was also significant, *F*(2,68) = 3.06, *p* = 0.05, $\eta_{p}^{2}$ = 0.08. This suggests that significant progress of the children from the PA group occurred during the intervention, and was maintained 6 months later.

#### Rapid Automatized Naming

The 2 × 2 ANOVA on RAN revealed a significant effect of testing time, suggesting an improvement of both groups in RAN, *F*(1,34) = 5.44, *p* = 0.03, $\eta_{p}^{2}$ = 0.14. A significant Group × Testing Time interaction was also observed, *F*(1,34) = 5.00, *p* = 0.03, $\eta_{p}^{2}$ = 0.13. The means suggest that the children trained in RAN improved more than the children trained in PA between T1 and T2. Moreover, the 2 × 3 ANOVA including T3 in the model also showed a significant Group × Testing Time interaction, *F*(2,68) = 3.09, *p* = 0.05, $\eta_{p}^{2}$ = 0.08. These two significant interactions indicate that the gains obtained by the children from the RAN group in RAN performance occurred during the intervention, and have remained stable 6 months later.

### Transfer Effects on Word Reading and Spelling

Transfer effects were analyzed with three-way repeated measures ANOVAs conducted on reading accuracy, reading speed, spelling accuracy, and spelling errors. Supplementary Figures 2 and 3 show word reading and spelling performances, and the proportions of different types of errors in spelling by group and testing time. **Tables 6** and **7** display mean performances and standard deviations.

**Table 6:** Descriptive statistics on reading and spelling measures by testing time and group.

Measures	Testing time	PA intervention (*N* = 18)		RAN intervention (*N* = 18)	
		*M*	*SD*	*M*	*SD*
Reading					
Regular word reading AC (raw score)^a^	T1	15.06	2.15	15.61	2.95
	T2	15.28	2.97	16.06	2.98
	T3	15.72	2.44	16.22	3.04
Irregular word reading AC (raw score)^a^	T1	5.17	3.52	6.56	4.09
	T2	6.06	3.57	8.00	4.50
	T3	9.39	3.52	9.22	4.62
Pseudoword reading AC (raw score)^a^	T1	14.89	3.18	15.78	3.10
	T2	15.67	2.30	15.61	2.99
	T3	15.44	2.09	15.39	3.57
Regular word reading RT (seconds)	T1	38.67	13.89	40.06	16.56
	T2	34.83	12.90	36.78	19.08
	T3	32.72	12.48	26.56	11.72
Irregular word reading RT (seconds)	T1	43.11	16.59	44.67	18.95
	T2	40.05	14.84	41.33	21.63
	T3	35.61	14.88	30.22	14.01
Pseudoword reading RT (seconds)	T1	38.78	12.46	44.11	16.32
	T2	37.94	16.09	33.83	8.02
	T3	31.39	10.51	28.22	9.94
Spelling					
Regular word spelling (raw score)^b^	T1	3.94	2.18	4.17	2.20
	T2	4.89	2.11	5.56	2.28
	T3	6.44	1.98	6.44	1.98
Irregular word spelling (raw score)^b^	T1	1.67	1.88	2.06	1.98
	T2	2.72	1.36	3.28	2.65
	T3	3.39	1.61	3.39	2.50
Pseudoword spelling (raw score)^b^	T1	5.50	2.20	5.72	2.19
	T2	6.94	2.18	7.00	2.30
	T3	7.50	2.41	6.83	2.26

*T (1–3), testing time; AC, accuracy; RT, response time. ^a^Single word reading task from the BALE, total correct responses out of 20. ^b^Word spelling task from the BALE, total correct responses out of 10.*

**Table 7:** Mean percentages of spelling errors by testing time and group.

Measures	Testing time	PA intervention (*N* = 18)		RAN intervention (*N* = 18)	
		*M*	*SD*	*M*	*SD*
Phonological	T1	27.73	10.86	29.28	19.11
errors	T2	15.96	13.43	28.47	18.01
	T3	16.85	13.43	28.70	24.44
Orthographic	T1	71.76	11.00	70.72	19.11
errors	T2	84.04	13.43	71.22	17.75
	T3	83.15	13.43	71.30	24.44
Non-responses	T1	0.51	1.49	0.00	0.00
	T2	0.00	0.00	0.31	1.31
	T3	0.00	0.00	0.00	0.00

*T (1–3), testing time.*

#### Reading Accuracy

The ANOVA including the two testing times (T1–T2) revealed an effect of testing time, indicating that children from both groups improved their accuracy in word reading over time, *F*(1,34) = 5.82, *p* = 0.02, $\eta_{p}^{2}$ = 0.15. However, no significant two-way (Group × Testing Time) and three-way (Group × Testing Time × Word Type) interactions were found (*p*s > 0.40). The interactions on this measure with three testing times entered in the model (T1–T2–T3) were neither significant (*p*s > 0.22).

#### Reading Speed

The ANOVA including the two testing times (T1–T2) revealed an effect of testing time on word reading speed, *F*(1,34) = 4.88, *p* = 0.03, $\eta_{p}^{2}$ = 0.13. The means show that the children from both intervention groups gained in reading speed between T1 and T2. There were no significant two-way (Group × Testing Time) and three-way (Group × Testing Time × Word Type) interactions (*p*s > 0.07). However, when considering the three testing times (T1–T2–T3), a significant Group × Testing Time interaction was found on reading speed, *F*(2,68) = 5.36, *p* = 0.01, $\eta_{p}^{2}$ = 0.14. Looking at the reported means, this interaction suggests that the children from the RAN group improved their word reading speed significantly more than the children trained in PA. These results suggest that the decrease in reading times occurred in the long run (T1–T2–T3).

#### Spelling Accuracy

The ANOVA including two testing times (T1–T2) showed a significant testing time effect on spelling accuracy, *F*(1,34) = 56.70, *p* < 0.001, $\eta_{p}^{2}$ = 0.63. After entering two (T1–T2) and three (T1–T2–T3) testing times in the model, no two-way (Group × Testing Time) or three-way (Group × Testing Time × Word Type) interactions reached significance (*p*s > 0.48).

#### Spelling Errors

Regarding the ANOVA including two testing times, no significant testing time effect and two-way interaction (Group × Testing Time) were found on the percentages of errors (*p*s > 0.27). There was a significant three-way interaction (Group × Testing Time × Error Type), *F*(2,68) = 4.48, *p* = 0.02, $\eta_{p}^{2}$ = 0.12. We wanted to know whether this interaction meant that there was a two-way interaction (Testing Time × Error Type) that varied across groups. To show it statistically, we ran the ANOVA separately for the PA group and the RAN group (split file). The results substantially differed in both groups regarding the Testing Time × Error Type interaction. This was significant for the PA group, *F*(2,68) = 13.32, *p* = 0.001, $\eta_{p}^{2}$ = 0.44, but not for the RAN group, *F*(2,68) = 0.09, *p* = 0.92, $\eta_{p}^{2}$ = 0.01. In the light of reported means, these results suggest that in the PA group, the percentage of phonological misspellings decreased significantly from pre-test to post-test, whereas the proportion of orthographic errors increased. In the RAN group, the different proportions of errors remained similar from pre-test to post-test. Interestingly, these results suggest a switch in the type of errors made by the children from the PA group following the intervention. While no change was revealed in the RAN group, the children from the PA group switched from phonological errors to orthographic errors. The three-way interaction was not significant anymore when entering three testing times (T1–T2–T3) in the model, *F*(2,68) = 2.07, *p* = 0.09, $\eta_{p}^{2}$ = 0.06, indicating that these transfer effects had not been maintained until the follow-up.

## Discussion

The present study aimed to further elucidate the relationship between PA, RAN, reading, and spelling in addressing three research questions. First, we wanted to determine whether RAN is independent of PA in training one competence and looking at the effect on the other. Second, we wanted to examine the specific causal influence of both oral abilities on word reading and spelling through the intervention design. Third, this study aimed to demonstrate the possibility of improving the RAN procedure with specific RAN-objects’ training. Thirty-six children were trained either in PA or in RAN over a period of 2 months, with 25-min lessons twice a week. They were assessed on PA, RAN, word reading, and word spelling, before, immediately after the intervention, and 6 months later. The results are summarized and discussed hereunder.

### PA and RAN As Independent Phonological Competencies

First of all, our results revealed specific effects of the two interventions. Indeed, children trained in PA improved in PA without improving in RAN, and children trained in RAN improved in RAN but not in PA from pre-test to post-test. Moreover, these effects were maintained 6 months later. These findings demonstrate the specific efficacy of a PA intervention on one side and of a RAN intervention on another side. This provides, with an intervention design, a new kind of evidence that PA and RAN constitute separate skills. This is opposed to the view of overlapping cognitive processes, advocated by authors who found that the RAN–reading relationship was mediated by PA (Bowey et al., 2005; Poulsen et al., 2015). Our results rather support the independence hypothesis where PA and RAN are considered as two separate predictors of reading outcomes. This assumption was initially proposed by Wolf and Bowers (1999) in the DDH. The DDH’s partisans defended RAN as a component apart from the phonological processing.

However, observing the relative independence of both oral abilities does not necessarily undermine the involvement of phonological processes during the RAN task. In line with Vellutino et al. (2004), PA and RAN could be independent skills, differentially affecting the diverse types of reading and spelling performance, and at the same time, influenced by a primary phonological factor. Further investigations are needed to better understand the common cognitive processes underlying both PA and RAN. But it seems that in our study, we trained distinct processes in each intervention: respectively the manipulation or the quick retrieval of the linguistic units of spoken language in verbal memory. It is worth nothing that these results cannot be explained by normal schooling. Although PA is often trained in kindergarten or in preschool classes, it was not trained in Grade 2. And RAN is usually not trained at all in schools.

### The Specific Transfer Effects of PA and RAN on Word Reading and Spelling

Our results revealed specific transfer effect of the interventions on reading and on spelling. On the one hand, training PA had no effect on word reading accuracy or word reading speed but was found to affect the *type of spelling errors* produced by the children. Compared to the children from the RAN group, children from the PA group committed significantly less phonological errors after the intervention. This kind of contrasted result confirms previous data showing that PA is more important in spelling development than in reading development (Landerl and Wimmer, 2008; Furnes and Samuelsson, 2011; Pan et al., 2011; Wolff, 2014). They are also in line with previous intervention studies demonstrating the efficacy of a phonemic training on spelling outcomes (Ball and Blachman, 1991; Schneider et al., 2000; Hulme et al., 2012). One reason could be that the phoneme-to-grapheme mappings involve mental phonological manipulations similar to the ones required by PA activities. On the contrary, grapheme-to-phoneme mappings are sustained by a visual support (i.e., the letters), unloading the working memory. Another reason in the present study could be related to the literacy acquisition stage and to the specificity of the orthographic system. As revealing by the high reading results at the pre-test, our French-speaking participants had already acquired some mastery and automaticity in decoding in Grade 2. Conversely, spelling abilities were still challenging. Yet, French has a quite opaque orthographic system in the sense of spelling, with a higher proportion of inconsistencies from phonology to spelling (79.1%) than from spelling to phonology (12.4%; Ziegler et al., 1996). Therefore, at this literacy acquisition stage, the influence of PA (i.e., ability to consciously segment and manipulate the code) could be of greater importance in spelling, which is still resting upon a high level of segmental abilities. Furthermore, it has to be specified that the significant decrease of the phonological errors committed by the children from the PA group was associated with an increase of the orthographic errors. As orthographic errors correspond to phonologically plausible spellings, this indicates a greater reliance on correct phoneme-to-grapheme mappings. Overall, this shift in the type of spelling errors could mean that a training of the PA skills benefits to the sub-lexical processes of spelling but not to the lexical processes (i.e., direct retrieval of the orthographic representations). Finally, the absence of transfer effects of the PA intervention on word reading skills is in line with the literature, which highlighted that PA trainings were more efficient prior to formal reading instruction or at the very beginning of learning to read (Bus and van Ijzendoorn, 1999).

On the other hand, the RAN intervention was found to be beneficial for the *word reading speed*. This is consistent with numerous correlational findings showing that RAN was a strong predictor of reading speed (van den Bos et al., 2002; Savage and Frederickson, 2005; Tan et al., 2005; Landerl and Wimmer, 2008; Araújo et al., 2015; Georgiou et al., 2016). However, this is the first time that the causal impact of RAN on reading speed is confirmed through an intervention design. In the other causal direction, a recent study (Wolff, 2014) showed that a reading training, which included speeded exercises, could indirectly enhance RAN. Those two pieces of evidence suggest a causal and reciprocal relationship between RAN and reading speed. Moreover, the present study allowed the comparison of RAN’s contribution to reading by means of accuracy and speeded measures of word reading. Our finding of a unique effect on the speed subcomponent supports RAN as an ability to automate the connections between perceptual and linguistic components in visually presented serial tasks (Norton and Wolf, 2012) that is of a great importance for the development of the automaticity of reading. This demonstrates conclusively that reading fluency is highly dependent on speed of lexical access. It also has to be mentioned that we found these significant results thanks to an intervention using objects matrices and no alphanumeric matrices. Moreover, our matrices included training items different from the test items. Importantly, this would mean that a procedural training of the rate of access to lexical whole units (i.e., objects) can give an advantage to reading speed and could be more efficient than a training of the access to a limited code (i.e., digits and letters) as it has mainly been proposed in previous studies. Finally, in line with the results from earlier studies (Wimmer and Mayringer, 2002; Georgiou et al., 2016), the RAN intervention had no impact on word spelling.

Altogether, these findings showed that both interventions impacted different aspects of the literacy skills. The PA intervention contributed to a decrease in the proportion of phonological errors committed during word spelling, whereas the RAN intervention contributed to improve the word reading speed. These very different and specific transfer effects bring additional support to the hypothesis of independence between PA and RAN.

### Effects and Practical Implications of the RAN Intervention

To the best of our knowledge, no research had shown a convincing improvement of RAN through a specific RAN training until now. Some authors showed no significant efficacy of the training (de Jong and Vrielink, 2004). Others gave evidence of the intervention efficacy, but they used flashcards rather than serial RAN (Fugate, 1997) or combined their intervention with a drill of other abilities, leading to confounding training effects (Conrad and Levy, 2011). Avoiding these biases, the current study demonstrated for the first time that RAN objects could be improved through carefully planned instruction.

Furthermore, the results revealed that the efficacy of our intervention occurred in the long run (i.e., 6 months after the intervention), and highlighted for the first time that such training was widely beneficial for reading achievement. These findings open up new perspectives for the prevention and remediation of reading disabilities. Indeed, although PA training is widely validated and commonly carried out at school or in speech therapy, no RAN training exists at this time. And, if traditional phonologically based interventions are appropriate to help single PA-deficit readers, it could be inappropriate or insufficient for children with a single RAN deficit. This kind of poor readers may benefit from additional or different objectives of intervention that best addresses their needs (Wolf, 1997). RAN interventions could be a new pedagogical way to reduce the incidence of reading problems among young children. They could also be a new tool to help children with specific RAN deficits and difficulties in reading fluency. The development of PA and RAN intervention could offer the possibility of providing appropriate tailored intervention to improve reading ability at the most, depending on the precise vulnerability of the child (RAN vs. PA).

Importantly, this is the first research to demonstrate direct and transfer effects of a RAN intervention using serial RAN objects. The RAN-objects’ task offers the advantage to be easily performed by young children contrary to alphanumeric RAN. Therefore, RAN-objects’ interventions have an even greater potential in prevention than RAN letters because children can perform RAN-objects’ matrices before beginning to learn to read.

More generally, our findings are innovative given that we have successfully improved children’s reading fluency by training them with an oral task (RAN objects), without having made them read at all. This finding opens up new perspectives for the remediation of poor reading fluency, complementary to current fluency interventions mainly based on repeated reading (for a review, see Stevens et al., 2016).

### Limitations and Future Avenues

There are several potential limitations to this study. A first concern is the poor effect sizes obtained on the interactions. They were mainly low and sometimes moderate. An explanation could be that the interventions were conducted at the end of Grade 2, when PA and RAN are already less robust in the prediction of learning to read. Another explanation could be the group size during the interventions at school. Indeed, the smaller the group is, the more frequent the opportunities for the children to practice the modeled skills (Kruse et al., 2015) and the stronger the effect sizes should be. Therefore, future studies are required to support our findings. First, the efficacy of the interventions should be stronger in younger children. As reported in the meta-analyses on PA training studies (Bus and van Ijzendoorn, 1999; Ehri et al., 2001), the earlier the start is, the better. For example, there is evidence of the powerful efficacy of a PA intervention on PA and later decoding skills in French-speaking kindergartners (Van Reybroeck et al., 2006b). Second, interventions could be more successful in disabled readers. With respect to PA instruction, Ehri et al. (2001) showed it gave a bigger boost in reading to at-risk children than it gave to normal readers. A recent study (Tilanus et al., 2016) also demonstrated that children with dyslexia made more progress than typical readers in grapheme–phoneme mappings, in both reading and spelling, following a phonic intervention. We do not have the same insight on RAN training. However, we could imagine that similarly, children with dyslexia could receive more benefits from a RAN intervention than typical readers.

Another limitation in this research is that there was no control group not receiving an intervention. Even though one experimental group was the control for the other, it would have been interesting to have had a no-trained group. Indeed, given that PA and RAN share at least partly some phonological processes, some part of the intervention effects could have not been grasped due to the comparison of improvement between both experimental groups.

Finally, the positive effects of the RAN intervention observed in this study on reading speed are limited to gains at the word level. We need other studies to determine the possible effects of such an intervention at the text level (e.g., including text reading fluency measures).

## Conclusion

The present study gave experimental support to the DDH, highlighting PA and RAN as separated cognitive processes, by means of an intervention design. Indeed, the children trained in PA improved in PA without improving in RAN, and the children trained in RAN improved in RAN but not in PA. The findings also emphasized the specific transfer effects of interventions in PA or in RAN on reading and spelling strategies. The PA intervention contributed to the sub-lexical processes of spelling (decreasing of the proportion of phonological errors), whereas the RAN intervention contributed to reading speed. Besides, this study provided evidence that the RAN performance can be enhanced through RAN-objects’ training focusing on rapid access to whole units in the mental lexicon. These results have determinant practical implications for teachers and speech therapists, RAN-objects’ training giving new hope for prevention and remediation of fluency reading problems among young children.

## Author Contributions

CVS performed the experiments and drafted the paper. CVS and MVR discussed the current literature, developed the research questions and hypotheses, designed the experiments, analyzed the data, critically revised the paper, and approved the version to be published.

## Conflict of Interest Statement

The authors declare that the research was conducted in the absence of any commercial or financial relationships that could be construed as a potential conflict of interest.
