# Supplementary material for: Phonological Awareness and Rapid Automatized Naming Are Independent Phonological Competencies With Specific Impacts on Word Reading and Spelling: An Intervention Study
Source: Front Psychol. 2018 Mar 13;9:320. doi: 10.3389/fpsyg.2018.00320 (PMC5859220; doi:10.3389/fpsyg.2018.00320)
Supplement: Supplementary file 1 [file Image_1.pdf]

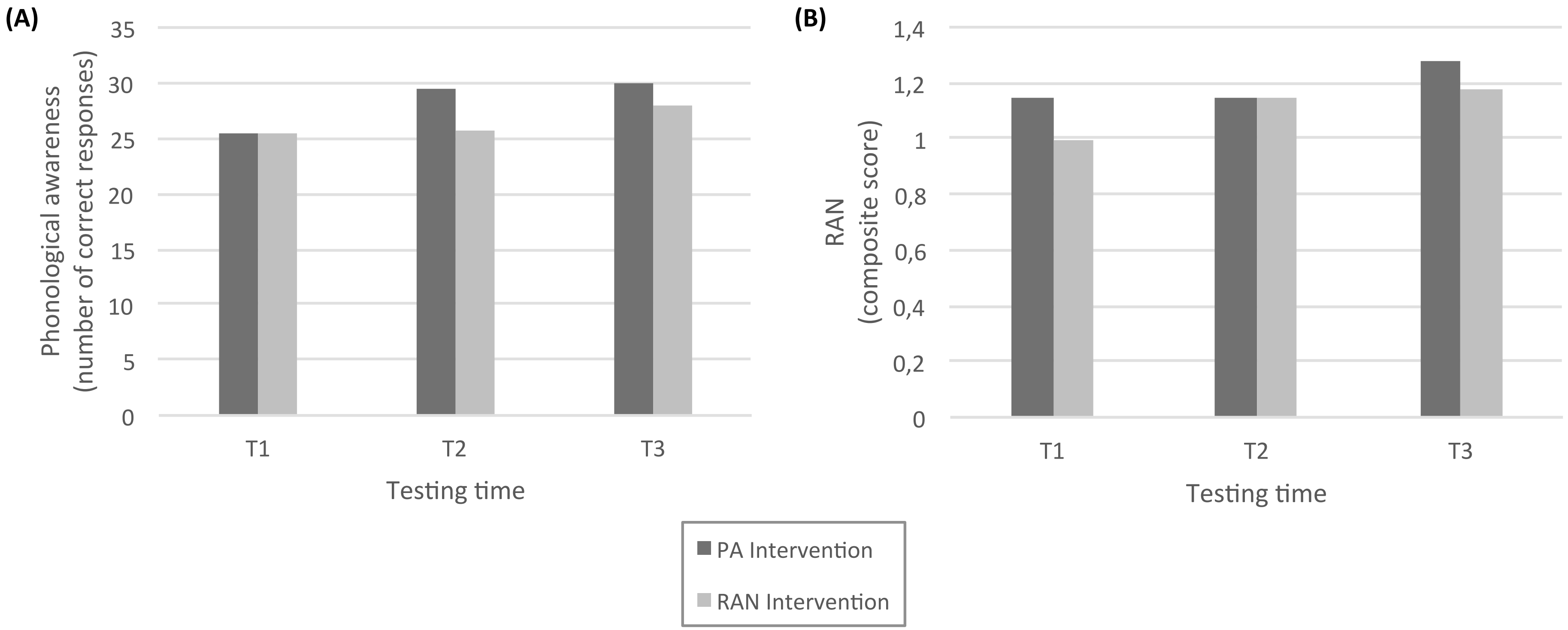

Supplementary Figure 1. Phonological awareness and RAN performances by testing time and group. **(A)** Total correct responses (out of 35 items) in phonological awareness. **(B)** RAN composite score (total correct responses divided by total naming time).

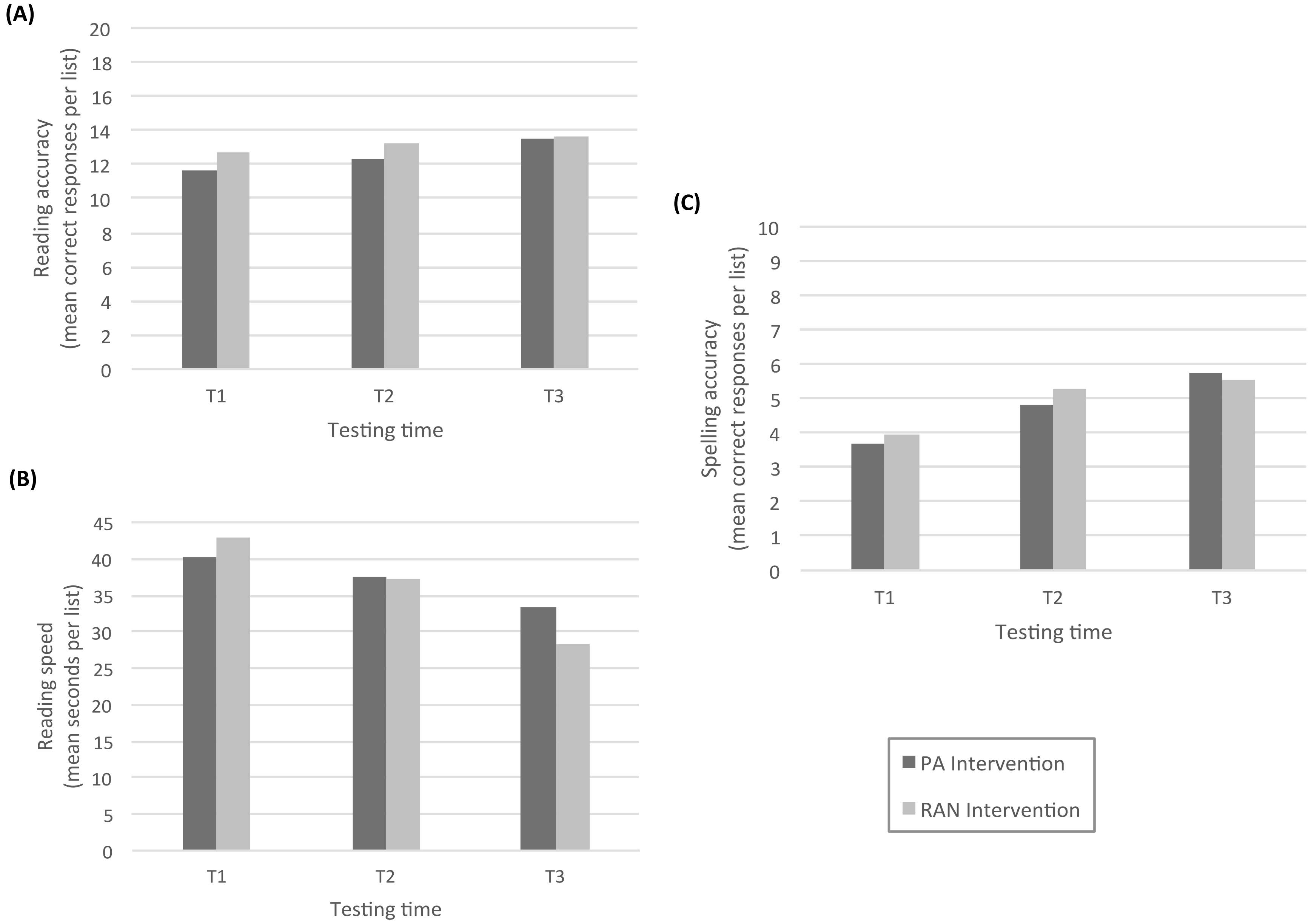

Supplementary Figure 2. Word reading and spelling performances by testing time and group. **(A)** Mean correct responses on the word reading lists (20 items per list). **(B)** Mean response times on the word reading lists (in seconds). **(c)** Mean correct responses on the word spelling lists (10 items per list).

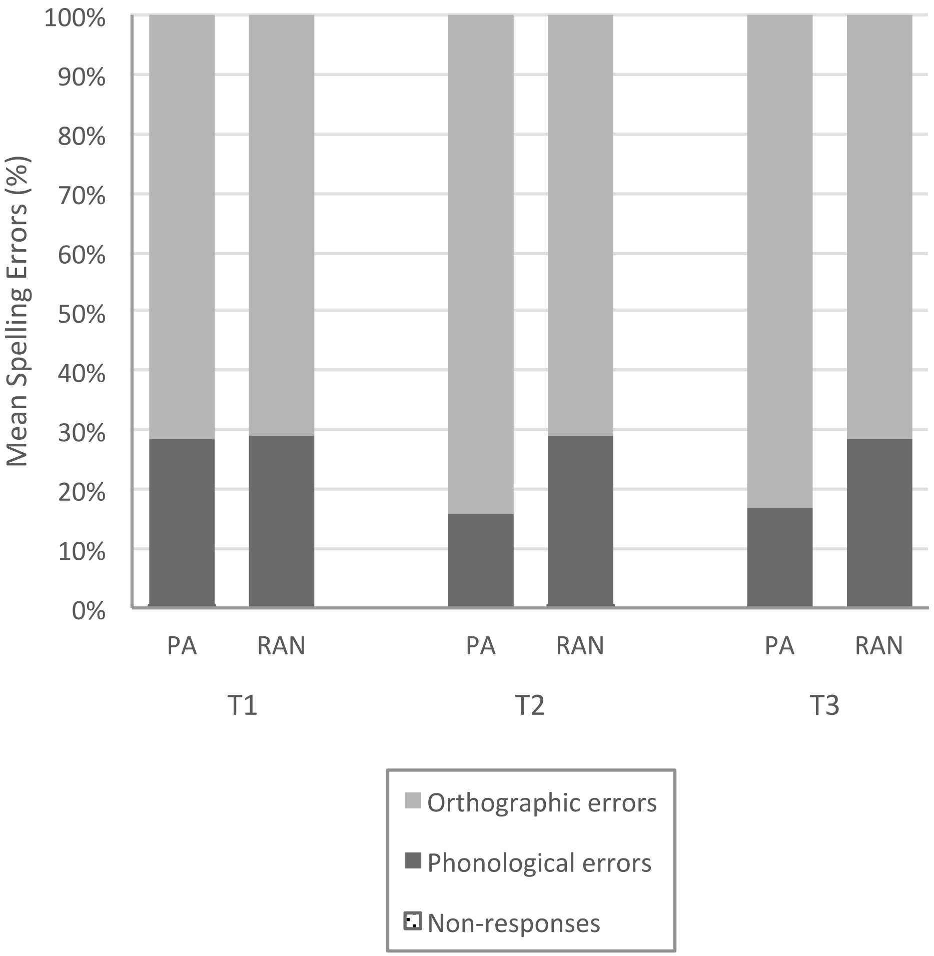

Supplementary Figure 3. Mean percentages of spelling errors by testing time and group.
